# DNA Barcodes for Species Identification in the Hyperdiverse Ant Genus *Pheidole* (Formicidae: Myrmicinae)

**DOI:** 10.1673/031.013.2701

**Published:** 2013-04-08

**Authors:** R.N. Ng'endo, Z.B. Osiemo, R. Brandl

**Affiliations:** 1 Department of Ecology, Animal Ecology Faculty of Biology Philipps-Universitaet Marburg, Karl-von-Frisch-Strasse 8 D-35032 Marburg, Germany; 2 Department of Zoology Faculty of Science Jomo Kenyatta University of Agriculture and Technology P.O BOX 62000-00200 Nairobi, Kenya

**Keywords:** *CO1,* DNA-barcoding, morphospecies, MOTU, taxonomy

## Abstract

DNA sequencing is increasingly being used to assist in species identification in order to overcome taxonomic impediment. However, few studies attempt to compare the results of these molecular studies with a more traditional species delineation approach based on morphological characters. Mitochondrial DNA Cytochrome oxidase subunit 1 (*CO1*) gene was sequenced, measuring 636 base pairs, from 47 ants of the genus *Pheidole* (Formicidae: Myrmicinae) collected in the Brazilian Atlantic Forest to test whether the morphology-based assignment of individuals into species is supported by DNA-based species delimitation. Twenty morphospecies were identified, whereas the barcoding analysis identified 19 Molecular Operational Taxonomic Units (MOTUs). Fifteen out of the 19 DNA-based clusters allocated, using sequence divergence thresholds of 2% and 3%, matched with morphospecies. Both thresholds yielded the same number of MOTUs. Only one MOTU was successfully identified to species level using the *CO1* sequences of *Pheidole* species already in the Genbank. The average pairwise sequence divergence for all 47 sequences was 19%, ranging between 0–25%. In some cases, however, morphology and molecular based methods differed in their assignment of individuals to morphospecies or MOTUs. The occurrence of distinct mitochondrial lineages within morphological species highlights groups for further detailed genetic and morphological studies, and therefore a pluralistic approach using several methods to understand the taxonomy of difficult lineages is advocated.

## Introduction

Identifying species can be difficult, often requiring specialized knowledge and thereby representing a limiting factor in biodiversity inventories (Monaghan et al. 2005). Therefore, based on the growing concern over the threats to biodiversity, recent publications have emphasized the need to accelerate the analysis of biodiversity (Brooks et al. 2004; Smith et al. 2005) either by using morphospecies (Hammond 1994; Oliver and Beattie 1996; Barratt et al. 2003; Krell 2004) or DNA-based methods (Floyd et al. 2002; Hebert et al. 2003a; Tautz et al. 2003; Blaxter 2004). Both morphological and molecular approaches have faced criticism (Pires and Marinoni 2010) due to the deficiencies encountered when using only a single approach for species identification (Knowlton 1993; Jarman and Elliot 2000; Rubinoff 2006; Rubinoff et al. 2006). The comparison of results obtained by various approaches can aid in overcoming methodological issues in species identification (Mengual et al. 2006; Smith et al. 2008). A further advantage of integrating molecular and morphological approaches (Dayrat 2005; Cardoso et al. 2009) is that it promotes taxonomic stability (Padial et al. 2010).

In this paper, a single gene, Cytochrome oxidase subunit 1 (*CO1*) (as proposed by Hebert et al. 2003a, b), was used for barcoding on morphologically pre-defined species (morphosecies) of the hyperdiverse ant genus *Pheidole* (Formicidae: Myrmicinae). The aim was to evaluate how DNA barcoding enables the definition of Molecular Taxonomic Units (MOTUs); Floyd et al. 2002; Blaxter et al. 2005), and then link the delineated MOTUs to the morphospecies in order to assess congruence success. This study focused on *Pheidole* samples from a region in the Brazilian Atlantic Forest because the region is considered as one of the “hottest hotspots” of biodiversity (Myers et al. 2000).

## Materials and Methods

### Research area

The study was carried out in the Rio Cachoeira Nature Reserve (25° 18′ 51″ S, 48° 41′ 45″ W) located near the city of Antonina, in the coastal region of the Brazilian state of Paranà. Specimens were obtained between June and September 2003 from leaf litter ants sampled across 12 study sites, representing four stages of secondary forest succession. There were three replicates (sites) for each succession stage, and the replicate sites of a particular succession stage were separated by a mean distance of 4 km (range = 1–6 km). At each study site, two 50 m transects (parallel, separated by 20 m) were established, and leaf litter samples were collected (1 m^2^) at 5 m intervals along these transects (10 sampling points for each transect). For more details on sampling methodology, see Bihn et al. (2008, 2010).

The landscape varies from littoral plains with isolated hills to the uplands of the Serra do Mar mountain range. Lowland and submontane forests originally covered this area, but these dense ombrophilous forests have been intensely exploited. Old growth forests remain only in the hillside regions. The resulting landscape mosaic consists of old growth forests and secondary forests in various stages of succession and pastures (Bihn et al. 2008, 2010).

### Definition of morphospecies

**Figure 1.**
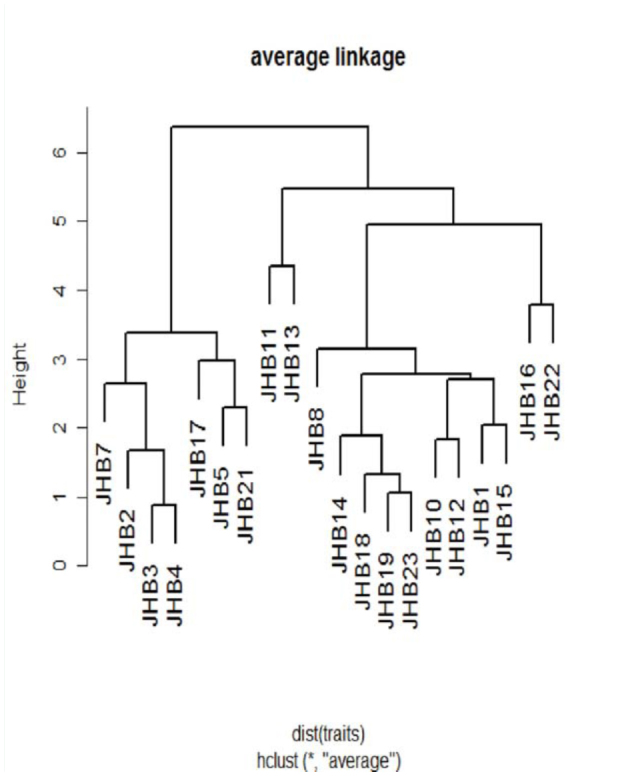
Hierarchical clustering using average linkage method on morphometric characters of the genus *Pheidole* from Rio Cachoeira Nature Reserve in the Brazilian Atlantic Forest, which defines 20 morphospecies. High quality figures are available online.

*Pheidole* specimens were identified to species with the key for neo-tropical species given by Wilson (2003). In cases where identification was not possible with this identification key (e.g., when major workers were not collected) or led to ambiguous results, ants were sorted into morphospecies using characters described by Wilson (2003). In addition, morphometric measurements were made to aid in the assignment of specimens into morphospecies (for details on the set of measurements taken and their definition, see Longino 2008). The morphometric characters used included: head width, distance between eyes, head length, anterior head length, scape length, mandible length, eye length, eye width, mesosoma length, promesonotal groove depth, propodeal spine length, femur length, tibia length, propodeal spiracle width, petiole width, and postpetiole width. The morphometric data were first standardize to mean = 0 and var = 1, and a hierarchical clustering of the morphospecies occurring in the Rio Cachoeira Nature Reserve was effected using the average linkage method (Figure 1).

### DNA extraction, amplification, and sequencing

Field collections were preserved in 95% ethanol until the time for DNA extraction. Specimens already examined and identified to be *Pheidole* morphospecies using morphological taxonomy were used for DNA extraction. Mitochondrial DNA was isolated for at least two minor workers from each morphospecies using the Qiagen DNeasy tissue extraction kit (Qiagen, www.qiagen.com) following the manufacturer's protocols. In cases where rare species were involved, DNA was extracted from a single individual, in which case either two legs or the whole individual was used.

Polymerase chain reaction (PCR) was conducted under the following reaction volumes: 2–4 µl DNA template, 2 µl in 10× PCR buffer, 1.6 µl of dNTPs in 10 mM concentra- tion, 1 µl of each primer in 10 mM concentration, 0.2 µl of Taq DNA polymerase, and distilled water for a total reaction volume of 20 µl. Reactions conditions included: initial denaturation at 95° C for 5 min; 33 cycles at 95° C (30 sec), 45–52° C at 40–48 sec (annealing time and temperature depended on primer used), and 72° C at 1 min; a final elongations at 72° C for 10 min reactions were done using an Eppendorf Thermal Cycler. Full-length sequences were amplified using primer pair LCO1490— GGTCAACAAATCAAAAGATATTGG and HCO2198— TAAACTTTCAGGGTGACCAAAAAATCA (Folmer et al. 1994). Primer pair LF1— ATTCAACCAATCATAAAGATATTGG and LR1— ATTTGAAGACCTACAGGTTTTTTAGT (Herbert et al. 2004a) was also used on specimens that were difficult to amplify using primers HCO/LCO. The two primer pairs gave the same length of base pairs. Products were visualized on a 2% agarose gel, and samples containing clean, single bands were purified using QIAquick PCR purification kit (Qiagen). The purified samples were sent for sequencing (AGOWA genomics, www.agowa.de), whereby the primers used in each case for amplification served as sequencing primers. All samples were sequenced in both directions, and the obtained sequences aligned using BioEdit version 7.0.9.0 (Hall 1999). The resultant fragments were approximately 658 base pairs (bp), and were identified as *CO1* fragments for the ant genus *Pheidole,* with BLAST procedure search in GenBank (Altschul et al. 1997) done between 2008 and 2009. After trimming, the aligned sequences were 636 bp long and free from gaps. A translation with the invertebrate mitochondrial code returned uninterrupted amino acid sequences. These observations support the conclusion that the sequences analyzed were mitochondrial DNA and not nuclear pseudogenes (Bensasson et al. 2001). All sequences were deposited in the GenBank under accession numbers JF825012–JF825054 and JF914928–JF914931.

### Phylogenetic analysis

Sequence divergences were calculated using the Kimura two parameter distance model (Kimura 1980), and the relationships between sequences were visualized by a Neighbor-Joining tree (Saitou and Nei 1987) using MEGA software version 4 (Tamura et al. 2007). A bootstrap test of phylogeny was effected by 100,000 replications and a similar random seed. To further infer relationships among the supposed morphospecies of *Pheidole,* phylogenetic analyses were performed using MrBayes version 3.1.1 (Huelsenbeck and Ronquist 2001), using the default value of four Markov chains and the General Time Reversible model. The Markov chain Monte Carlo length was 2,000,000 generations, sampled every 100 generations (burn-in = 4,500). Convergence of the chains was confirmed in the two runs by the examination of the average standard deviation of split frequencies, which in the present study had approached 0.007. Bayesian posterior probabilities were estimated as the proportion of the trees sampled after the burn-in that contained each of the observed bipartitions (Rannala and Yang 1996; Larget and Simon 1999). The phylogenetic tree was rooted using two species of the tribe Pheidolini, *Aphaenogaster texana* and *Messor julianus.*

The MOTU delineation from the *CO1* sequences relied on two aspects. First, individuals were considered to be the same MOTU if sequences from the same morphospecies clustered together in the phylogenetic tree. A MOTU was thus defined as the least inclusive terminal groups (i.e. closest to the tips). Second, sequence clusters with a mean divergence value less than or equal to a threshold of 2% and 3%, as proposed by Herbert et al. (2004b), were considered as MOTUs. In this case, if sequences from two different morphospecies formed the same cluster, they only qualified to be a single MOTU if their mean sequence divergence was below or equal to thresholds 2% and 3%.

Match success of the 47 sequences was further examined in relation to the *CO1* sequence of species in the genus *Pheidole* already present in the *CO1* Genbank library (NCBI, GenBank, http://www.ncbi.nlm.nih.gov/) (searches done between 2008 and 2009). In cases where the match success was above 95%, the species name for that MOTU was allocated. To establish the distribution of genetic divergence and positioning of MOTUs in relation to *Pheidole* species from other regions, all *CO1* sequences (genus *Pheidole)* that contained 640 or more bp (sequences retrieved on 2 March 2011) were extracted from the Genbank. A total of 141 sequences were obtained and combined with 47 sequences from this study for further alignment. The final set of 188 sequences was trimmed to 636 bp, and a histogram and a Neighbor-Joining tree were constructed (tree in Figure 4) using Kimura two parameter distance (Kimura 1980). This was implemented using data application Package ape (Paradis et al. 2004) available in R (R Development Core Team 2009).

## Results

**Figure 2.**
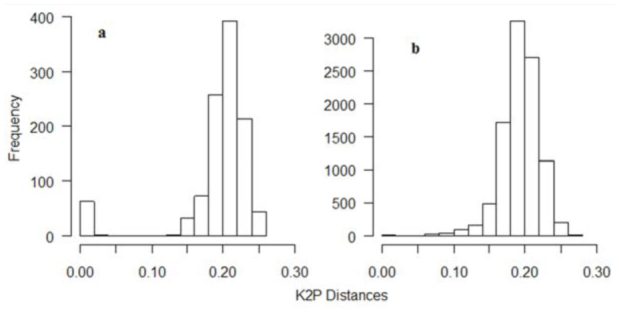
Distribution of pairwise distances of ant genus *Pheidole* calculated using Kimura two-parameter model (Kimura 1980) among (a) 47 Cytochrome oxidase 1 (CO 1) sequences from Rio Cachoeira Nature Reserve in Brazil and (b) 141 CO1 sequences from the Genbank. The 47 sequences have a peak near zero and another between 18% and 24%, while the 141 sequences have a major peak between 16% and 24% of sequence divergence. High quality figures are available online.

This study produced a final aligned 636 bp fragment, characterized with no gaps for all the 47 sequences. Sequences were heavily AT biased (especially in the third codon position), as is expected in insect mitochondrial DNA (Crozier and Crozier 1993; Table 1). The average pairwise sequence divergence of all 47 sequences was 19%, ranging from 0–25% (Figure 2a). The distribution of Kimura two parameter distances for 47 sequences showed one peak near zero and another between 18 and 24%, while the 141 *CO1* sequences from Genbank had a peak between 16 and 24% of sequence divergence (Figure 2b).

**Table 1.**
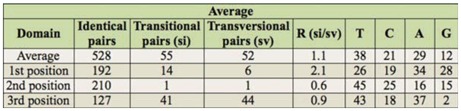
Sequence statistics for the 47 specimens used in the analysis of ant genus *Pheidole.*

**Figure 3.**
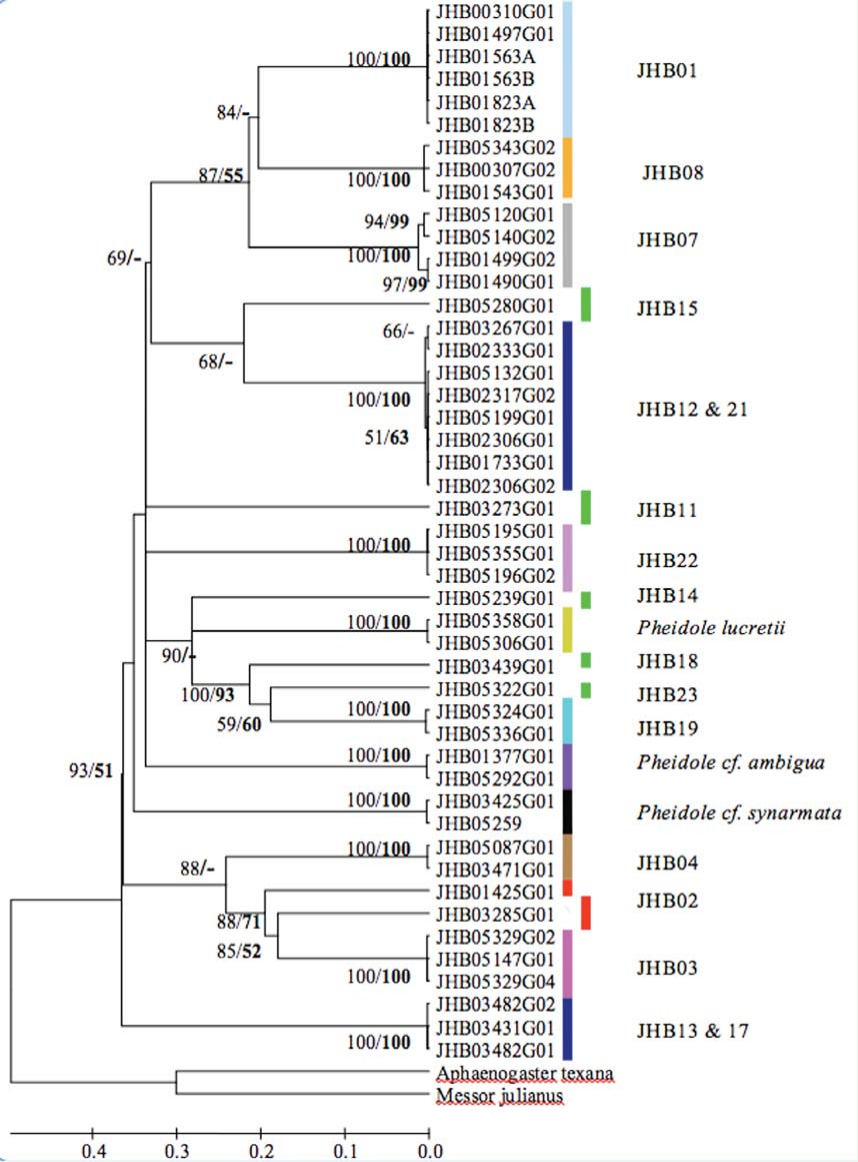
Linearized Bayesian tree of the ant genus *Pheidole* from Rio Cachoeira Nature Reserve in the Brazilian Atlantic Forest, which defines 19 MOTUs. The clustering of individual sequences in the tree indicates the membership of each MOTU. MOTUs were inferred from a tree dependent clustering process, coupled with thresholds of 2%. Each colored bar represent a MOTU. The five green bars represent cases where only one individual was sequenced, two red bars indicate possible cryptic taxa, and the other two dark blue bars indicate MOTUs with shared taxa. The three names in front of the bars represent *Pheidole* species whose names were assigned based on morphological taxonomy, and the numbers preceded by JHB represent the different morphospecies. Posterior probability values for Bayesian tree and bootstrap support values for Neighbor-joining tree (in bold) above 50% are indicated on the nodes. A dash (-) indicates bootstrap values below 50%. High quality figures are available online.

**Figure 4.**
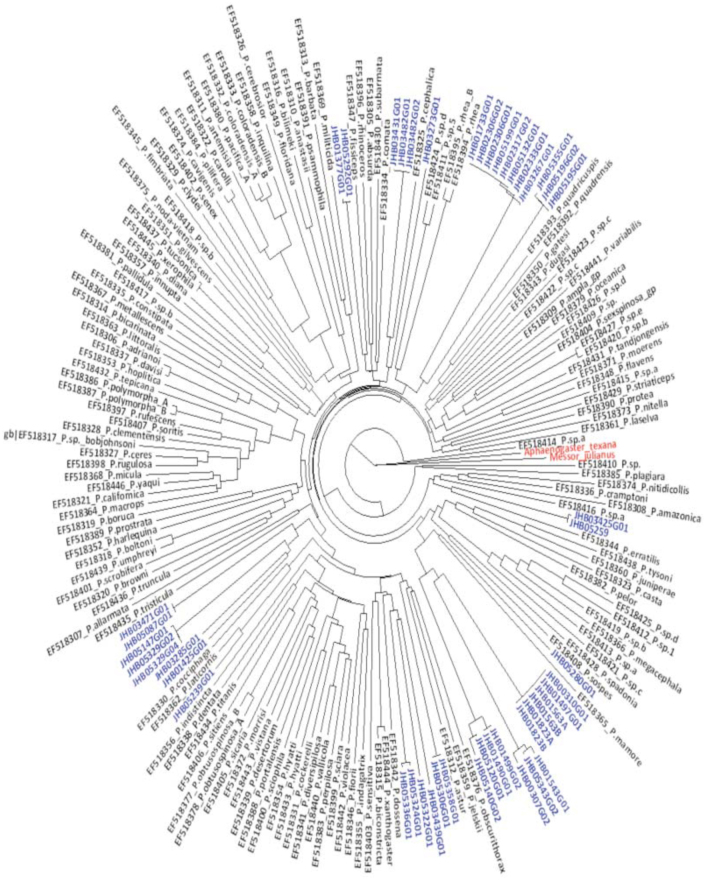
A combined phylogeny of *CO1* sequences all from the genus *Pheidole* from the Genbank, and 47 sequences (taxa in blue) from Rio Cachoeira Nature Reserve. The 47 sequences formed distinct clusters in relation to those from the Genbank. The tree is rooted using the taxa in red. High quality figures are available online.

Through morphology-based taxonomy, 20 morphospecies were identified (Figure 1), three of which were allocated species names (see Figure 3). DNA analysis identified 19 MOTUs, 15 of which matched with the morphospecies—about 79% match success. Fortysix sequences showed a matching of below 95% with *CO1* sequences from the Genbank, which ranged from 83 to 87%. Only the sequence for morphospecies JHB14 showed a 96% match with the Genbank sequence of *Pheidole laticornis.* A phylogeny containing a combination of 141 *CO1* sequences of *Pheidole* species from the Genbank and our 47 sequences showed distinct clusters for the MOTUs (Figure 4, taxa in blue). Specimens with only one sequence were regarded as a MOTU using the 2% or 3% criterion. In four MOTUs, morphological taxonomy did not match the results of the DNA-based approach.

The clustering of the 47 *CO1* sequences in NJ and Bayesian trees showed congruence with most morphospecies groups, with most nodes immediately below (i.e., defining) clusters showing a bootstrap support and a posterior probability of 100 (Figure 3). Divergences between sequences making up different *CO1* clusters (MOTUs) were far higher than divergences within a cluster of MOTUs (11-fold higher), with average Kimura two parameter divergences within and between clusters being 1.8% and 20% respectively (Figure 2). Exceptions occurred where deep sequence divergences were apparent between individuals identified as the same morphospecies (the two red bars in Figure 3, JHB03285G01 and JHB01425G01). These have a mean sequence divergence of 12.6%, a divergence 6–4 fold higher than the 2% and 3% thresholds respectively, which were used to allocate individuals into their respective clusters.

## Discussion

The results indicated that *CO1* sequences showed promising success in allocating morphologically pre-defined individuals into distinct *Pheidole* MOTUs. Most sequences clustered into cohesive, well-differentiated groups, most of which showed congruence with the predefined morphospecies. The majority of nodes immediately defining the clusters showed remarkably high levels of nodal support. Furthermore, most clusters remained distinct as sample sizes increased during the progress of the work, an indication that such groups included distinct *CO1* lineages rather than scattered sequence variation (Hajibabaei et al. 2006). In addition, sequence divergence in *CO1* mitochondrial DNA within MOTU (clusters) was usually much lower than 2%, whereas divergence between the clusters was often greater, but remained within the range of divergences between *CO1* sequences of *Pheidole* species from the Genbank. This result is in general agreement with empirical levels of divergence found between species in barcoding studies (Hebert et al. 2003b). These aspects strengthen the fact that most of the identified morphospecies were indeed distinct lineages (Wiens and Penkrot 2002).

A total of 20 morphospecies were recovered using morphological characters, whereas 19 MOTUs were recovered using barcodes. The diversity estimates (MOTU) using threshold values of 2% and 3% were similar to the diversity estimate based on morphological characters. In cases where the 2–3% MOTU and the morphological estimation of a species differed, either different morphospecies clustered to form the same MOTU (e.g., as was the case with the two MOTUs represented by the dark blue bars in Figure 3) or sequences from the same morphospecies showed deep sequence divergence (e.g., MOTUs represented by two red bars in Figure 3) and were thus allocated as different MOTUs.

Morphological reexamination of the two MOTUs that shared morphospecies (i.e., JHB13 and JHB17, JHB12 and JHB21; MOTUs indicated with dark blue bars in Figure 3) revealed significant differences in morphometric characters between the shared species, as evidenced by the hierarchical clustering (Figure 1). The grouping into one MOTU may be due to incomplete lineage sorting or even mitochondrial introgression (Herbert and Gregory 2005; Meyer and Paulay 2005). Incomplete lineage sorting or gene introgression could be possible in taxa with shared sequences/haplotypes because the species occurred in the same locality. This explanation is further reflected by their low mean sequence divergence (below 0.02) and high bootstrap support for their respective clusters. On the contrary the two MOTUs representing possible cryptic taxa (two red bars; JHB02 in Figure 3) had high mean sequence divergence and their clustering was not strongly supported, thereby qualifying them to possibly be different species. Further reevaluation of the four MOTUs either for introgression or cryptic diversity using mitochondrial DNA was, however, hampered by the limited samples.

The results further revealed very low success when matching MOTUs with *Pheidole* species already in a *CO1* library. After integrating the 47 sequences with those from the Genbank, clusters of the MOTUs remained stable within the phylogeny, with only four MOTUs forming monophyletic clusters with the species from the Genbank (Figure 4). The species name for MOTU JHB05239G01 was allocated as *Pheidole laticornis* based on the set criteria of allocating species names in this study and others (Meier et al. 2006). Overall, it was difficult to allocate species names to MOTUs based on *CO1* sequences in the library. This observation does not imply that *CO1* cannot be used in species identification as barcodes, but for the MOTUs in this study, other strategies will be necessary. The low success in matching these MOTUs with Genbank sequences was likely because only a few of the more than 600 described species of *Pheidole* are included in Genbank. Also, there are many undescribed ant species in the neo-tropical region, and there are suggestions that many species in the Mata Atlantica may be endemic (R. Brandl, personal communication).

Congruence success between the two species identification approaches was not very high, which may be attributed to the criteria applied in delimiting MOTUs. For instance, threshold approach is vulnerable to both false positives and false negatives (Meyer and Paulay 2005). Regardless of such shortcomings in both morphological taxonomy and DNA barcoding (DeSalle et al. 2005; Pires and Marinoni 2010), the low congruence success does not compromise their effective use for species identification (Smith et al. 2005); on the contrary, either approach helps to illuminate taxonomic assignments in need of further scrutiny (Herbert and Gregory 2005; Padial et al. 2010). Such scenarios call for a more thorough morphological and *CO1* diversity survey among the members of the involved taxa. Moreover, in cases of introgression, the analysis of a rapidly evolving nuclear sequence, such as the internal transcribed spacer region of the ribosomal repeat, will aid taxonomic resolution (Herbert et al. 2003a). However, this study did not manage to employ other molecular markers for species delimitation, and was only limited to mitochondrial DNA.

Five rare morphospecies were represented with only a single sequence (MOTUs represented by the green bars in Figure 3) due to the used sampling techniques and were coded by the 2% and 3% criterion as MOTUs. A previous study on DNA barcoding of ants using these thresholds (Smith et al. 2005) recommended that only by sampling multiple individuals from supposed species, or MOTUs, will inter-specific variation be properly assessed. Otherwise, it is impossible to test the hypothesis of species-level monophyly (Funk and Omland 2003) and could lead to biodiversity overestimation. This is a valid concern in an analysis of MOTUs from inventories of hyperdiverse groups such as ants, which often include many taxa known only from single individuals (Fisher 1999; Longino et al. 2002). Morphological identification of such rare species also calls for the use of multiple individuals in order to assess the conformity in taxonomic characters within individuals of a given taxa. With additional inventories in the future, many of these rare species will be represented in collections by more specimens.

The aim of this study was to investigate the efficacy of DNA barcoding in delimiting predefined species. The results provide an example of the complementarity with which DNA barcoding can be applied together with a more conventional morphological approach, without competing or replacing the latter approach (Hebert and Gregory 2005). Moreover, thresholds of 2% and 3% proved to be effective in delineating species in the genus *Pheidole.* Despite the shortcoming in match success, this study demonstrated that diversity estimates using *CO1* MOTU together with morphological taxonomy offer a means to map the occurrence of ant species that still wait to be formally described and included into keys for identification.
